# Genetic Engineering and Enrichment of Human NK Cells for CAR-Enhanced Immunotherapy of Hematological Malignancies

**DOI:** 10.3389/fimmu.2022.847008

**Published:** 2022-04-07

**Authors:** Maren Soldierer, Arthur Bister, Corinna Haist, Aniththa Thivakaran, Sevgi Can Cengiz, Stephanie Sendker, Nina Bartels, Antonia Thomitzek, Denise Smorra, Maryam Hejazi, Markus Uhrberg, Kathrin Scheckenbach, Cornelia Monzel, Constanze Wiek, Dirk Reinhardt, Naghmeh Niktoreh, Helmut Hanenberg

**Affiliations:** ^1^ Department of Pediatrics III, University Children’s Hospital Essen, University Duisburg-Essen, Essen, Germany; ^2^ Department of Otorhinolaryngology, Head & Neck Surgery, University Hospital Düsseldorf, Heinrich Heine University, Düsseldorf, Germany; ^3^ Department of Experimental Medical Physics, Heinrich Heine University, Düsseldorf, Germany; ^4^ Institute for Transplantation Diagnostics and Cell Therapeutics, Heinrich-Heine University, Düsseldorf, Germany

**Keywords:** human NK cells, chimeric antigen receptor, genetic engineering, lentiviral vectors (LVS), adoptive cellular immunotherapy, transduction

## Abstract

The great clinical success of chimeric antigen receptor (CAR) T cells has unlocked new levels of immunotherapy for hematological malignancies. Genetically modifying natural killer (NK) cells as alternative CAR immune effector cells is also highly promising, as NK cells can be transplanted across HLA barriers without causing graft-versus-host disease. Therefore, *off-the-shelf* usage of CAR NK cell products might allow to widely expand the clinical indications and to limit the costs of treatment per patient. However, in contrast to T cells, manufacturing suitable CAR NK cell products is challenging, as standard techniques for genetically engineering NK cells are still being defined. In this study, we have established optimal lentiviral transduction of primary human NK cells by systematically testing different internal promoters for lentiviral CAR vectors and comparing lentiviral pseudotypes and viral entry enhancers. We have additionally modified CAR constructs recognizing standard target antigens for acute lymphoblastic leukemia (ALL) and acute myeloid leukemia (AML) therapy—CD19, CD33, and CD123—to harbor a CD34-derived hinge region that allows efficient detection of transduced NK cells *in vitro* and *in vivo* and also facilitates CD34 microbead-assisted selection of CAR NK cell products to >95% purity for potential clinical usage. Importantly, as most leukemic blasts are *a priori* immunogenic for activated primary human NK cells, we developed an *in vitro* system that blocks the activating receptors NKG2D, DNAM-1, NKp30, NKp44, NKp46, and NKp80 on these cells and therefore allows systematic testing of the specific killing of CAR NK cells against ALL and AML cell lines and primary AML blasts. Finally, we evaluated in an ALL xenotransplantation model in NOD/SCID-gamma (NSG) mice whether human CD19 CAR NK cells directed against the CD19+ blasts are relying on soluble or membrane-bound IL15 production for NK cell persistence and also *in vivo* leukemia control. Hence, our study provides important insights into the generation of pure and highly active allogeneic CAR NK cells, thereby advancing adoptive cellular immunotherapy with CAR NK cells for human malignancies further.

## Introduction

Cancer immunotherapy, entitled by *Science* as the breakthrough of the year 2013 (1), continues to grow exponentially, and harnessing the power of cellular therapies has contributed significantly to this progress. Although adoptive cellular therapies had been used in the past as rather experimental treatments for patients after stem cell transplantation, with late-stage disease and/or metastatic solid tumors (2–4), the immense potential of cellular immunotherapies became obvious since 2010 through the introduction of second-generation chimeric antigen receptor (CAR) T cells in clinical phase I/II studies (5, 6). Since then, CAR T cells have overcome many limitations of autologous adoptive immunotherapies, which previously were using tumor-infiltrating lymphocytes (TILs) isolated from malignant tissues, expanded *in vitro*, and reinfused into the patients (7). In contrast, CAR T cells do not require extended sampling of tumor tissues, as the patients’ autologous circulating T cells are engineered *ex vivo* to recognize tumor-associated antigens, thus making CAR T cell therapy principally applicable for both solid tumors and hematological malignancies (8).

The great clinical success of CAR T cells in early trials for relapsed or refractory hematological malignancies of the B cell lineage has already resulted in the approval of five CAR T cell products, targeting CD19-positive leukemia and lymphoma (Kymriah^®^, Yescarta^®^, Tecartus^®^, and Breyanzi^®^) or B cell maturation antigen (BCMA)-positive multiple myelomas (Abecma^®^) (6, 8–10). These second-generation CAR constructs typically consist of extracellular antibody-derived sequences that determine the specificity and two intracellular T cell signaling units, usually the zeta chain of the CD3 complex and either CD28 or 4-1BB/CD137 as co-stimulatory domains. Thus, CARs on competent immune effector cells can recognize surface target antigens independent of any human leukocyte antigen (HLA) constellation and then kill the target cells (6). However, to avoid allogeneic graft-versus-host disease (GvHD), the CAR T cell products need to be generated in autologous settings, which results in very expensive and long manufacturing pipelines. In addition, a high number of clinical trials have reported severe adverse events following autologous CAR T cell treatment, such as life-threatening cytokine release syndrome (CRS) or neurotoxicity (6).

Natural killer (NK) cells are professional immune effector cells of the innate immune system that can recognize and lyse their target cells in a non-antigen-specific manner, thereby enabling them to effectively detect and eliminate malignant cells that have escaped the T cell immune surveillance (11, 12). Most importantly, as NK cells are not HLA-restricted and when transplanted do not cause acute or chronic GvHD, they can readily be administered to HLA-mismatched patients and have, when obtained from healthy donors, significantly shorter manufacturing periods (13). In addition, since large numbers of immune effector cells are required for successful therapeutic transplantation, and leukemia patients often have limited numbers of leukocytes due to their heavy pretreatment regimens, the potential to use allogeneic CAR NK cells of healthy donors for therapeutic infusions would be a major advantage over autologous CAR T cells and allows *off-the-shelf* usage of pre-manufactured products (11). Notably, NK cells can readily be genetically modified with lentiviral vectors (14) and the classical second-generation CARs with either CD28 or 4-1BB signaling domains function well in NK cells and confer additional antitumor effects (11). In preclinical xenograft murine models, the activity of CAR NK cells against malignant cells was similar to that of CAR T cells, albeit with less cytokine release and better overall survival rates, at least for ovarian cancer (15) and acute lymphoblastic leukemia (ALL) (16). Importantly, the first clinical phase I/II trial with CD19 CAR NK cells for the treatment of relapsed or refractory CD19-positive leukemias (NCT03056339) reported high response rates and no treatment-associated occurrence of CRS, neurotoxicity, or GvHD (17). Hence, CAR NK cells for specific target antigens appear to be safe and could potentially be used as *off-the-shelf products*, thus drastically shortening the production time and lowering the costs of CAR-based cellular cancer therapeutics (18).

Compared to a large number of clinical studies with CAR T cells, CAR NK cell therapy development is clearly lagging behind (9). Most of the delay can be attributed to two major problems, the comparably low transduction rates and the challenges in the large-scale genetic engineering of primary human NK cells from healthy donors (19). In contrast to human T cells, which can readily be transduced with lentiviral vectors using the VSV-G glycoprotein as envelope pseudotype (20, 21), NK cells do not express sufficient amounts of the cellular surface molecules of the LDL receptor family that serve as entry receptors for VSV-G (21). Therefore, different envelope pseudotypes are necessary for the efficient entry of the lentiviral particles (22). In their seminal publications, Girard-Gagnepain et al. demonstrated that two constructs derived from the envelope protein of the baboon endogenous virus, BaEV-Rless, and BaEV-TR, are optimally suited for genetically modifying resting hematopoietic cells (14) and also human primary NK cells with lentiviral vectors (21, 23). The most efficient and reliable large-scale expansion of primary human NK cells up to date can be performed by using genetically engineered cells of the AML cell line K562 as irradiated feeder cells that express the 4-1BB ligand, membrane-bound IL15, and/or IL21 (17, 19, 24, 25). However, using leukemic K562 cells for stimulation of cellular products that will be injected into patients is not ideal.

In this study, we intended to establish the transduction of primary human NK cells derived from peripheral blood in a feeder-cell-free and CliniMACS Prodigy™-compatible system (26) using the NK MACS medium from Miltenyi Biotec and also addressed several issues in genetic engineering of NK cells, including optimized transgene expression, alternative lentiviral pseudotypes, and transduction enhancers. We also established a methodology for the enrichment of human CAR NK cells and developed an assay system to functionally test the CAR-specific cytotoxic activity against leukemic cell lines and primary AML blasts *in vitro.* Finally, we addressed conditions for the prolonged survival of CAR NK cells *in vivo* in immunodeficient NOD/SCID-gamma (NSG) mice with B-cell precursor ALL.

## Material and Methods

### Cell Culture

The AML cell lines MOLM-14, NOMO-1, CMK, and THP-1 and the ALL cell lines REH and BV-173 were obtained from the DSMZ (Braunschweig, Germany) and cultured in RPMI-1640 GlutaMAX™ medium supplemented with 10% fetal bovine serum (FBS) and 1% penicillin and streptomycin (P/S; all from Thermo Fisher Scientific, Waltham, MA, USA), referred to as complete RPMI-1640. Human embryonic kidney cells (HEK293T, DSMZ) were cultured in Dulbecco’s Modified Eagle Medium (DMEM) GlutaMAX™ supplemented with 10% FBS and 1% P/S, referred to as complete DMEM.

### Isolation and Culture of Human NK Cells

Primary human NK cells were isolated from peripheral blood of healthy donors, for which they gave written informed consent according to the protocol (#2019-623) approved by the local ethics committee (Heinrich Heine University, Düsseldorf, Germany). Ficoll density gradient centrifugation (Ficoll-Paque Plus; Cytiva Europe, Freiburg, Germany) was performed to collect peripheral blood mononuclear cells (PBMCs). NK cells were then enriched by immunomagnetic negative selection using MojoSort™ Human NK Cell Isolation Kit (BioLegend, San Diego, CA, USA) according to the manufacturer’s instructions. NK cell purity and phenotype were determined by flow cytometry (MACSQuant^®^ Analyzer 10, Miltenyi Biotec, Bergisch Gladbach, Germany) on days 0, 7, and 14, using fluorochrome-conjugated antibodies against CD3, CD33, CD56, and CD94 (all REAfinity™ clones from Miltenyi Biotec). NK cells were activated and expanded in NK MACS^®^ Medium (Miltenyi Biotec) supplemented with 5% heat-inactivated human AB serum (Sigma-Aldrich, Darmstadt, Germany), 1% P/S, 500 IU/ml of IL2 (Proleukin, Novartis, Basel, Switzerland), and 10 ng/ml of IL15 (Miltenyi Biotec) at a concentration of 1–2 million cells/ml.

### Lentiviral Expression Constructs

All CAR constructs were optimized for human codon usage and synthesized by GeneArt (Thermo Fisher Scientific) or BioCat (Heidelberg, Germany). The CARs were co-expressed *via* a T2A site with the monomeric tag blue fluorescent protein (BFP from Evrogen, Moscow, Russia) under the control of the myeloproliferative sarcoma virus (MPSV) promoter (27) and equipped with our CD34-derived hinge C6 (28), CD28 transmembrane, and co-stimulatory domains as well as the CD3 zeta-chain unit (27). The CD19, CD33, CD123, and epidermal growth factor receptor (EGFR) (clone cetuximab/C225, from now on referred to as Cetux) CARs were previously described (20, 27–29). In the ALL xenograft model, CD19 CARs were co-expressed with codon-optimized soluble human IL15 or human IL15 tethered to the IL15 receptor α-chain (IL15-IL15R). Both IL15 cDNAs used a codon-optimized CD8 leader peptide. The codon-optimized firefly luciferase/enhanced green fluorescent protein (EGFP) fusion (LucEG) (20) and the EGFP/neomycin resistance fusion (EGN) (27) lentiviral expression vectors were previously published. The MPSV, hPGK (human phosphoglycerate kinase 1), modified spleen focus-forming virus (SFFV), and short EF1α (elongation factor 1-α) promoter constructs were also previously published (27). The wild-type SFFV was newly introduced in the pCL6EGNwo vector (27). The optimized EF1α promoter was generated from the long EF1α construct with the wild-type SD and acceptor sites (27) by deleting ~600 bp of the natural intron and mutating an open reading frame (sequences are available upon request).

### Production of Lentiviral Particles

The production of lentiviral particles in HEK293T cells was performed on 10-cm dishes as previously described (20, 30). For pseudotyping, the following envelopes were used: the vesicular stomatitis virus glycoprotein (VSV-G, 6 µg per transfection), the codon-optimized baboon endogenous virus lacking the R-peptide (BaEV-Rless, 1 µg per transfection), the codon-optimized BaEV surface and transmembrane units fused to the cytoplasmic units of the amphotropic murine leukemia virus (aMLV; BaEV-TR, 1 µg per transfection), the codon-optimized gibbon ape leukemia virus (GALV) surface unit fused to the transmembrane and cytoplasmic units of aMLV (GALV-TM, 1 µg per transfection), or the previously published (31) feline endogenous virus envelope fused to the cytoplasmic units of aMLV (RD114-TR, 6 µg per transfection). Twenty-four hours after transfection, the culture medium was replaced with 10 ml of IMDM (Sigma-Aldrich) supplemented with 10% FBS, 1% P/S, and 1% l-glutamine (Thermo Fisher Scientific). Cell supernatants containing viral particles were harvested 48 h after transfection, filtered through a 0.45-µm filter, and either used directly or after concentration (5-fold) at 10,000 × *g* for 2 h at 4°C. Lentiviral supernatants were used fresh or stored at −80°C until usage.

### Transduction of Human NK Cells

After 7 to 10 days of expansion in a complete NK MACS medium, primary human NK cells were transduced with lentiviral particle-containing supernatants. For the envelope, promoter, and entry enhancer testing, 1 to 1.5 × 10^5^ NK cells per well were transduced in flat-bottom 96-well plates in working volumes of 100 µl with serially diluted lentiviral particles encoding EGN. When RetroNectin™ (TaKaRa Bio Inc., Otsu, Japan; from now on referred to as Retronectin) was used as transduction enhancer, non-treated 96-well plates were coated either overnight at 4°C or for 2 h at 37°C prior to use (32, 33). In contrast, Vectofusin^®^-1 (Miltenyi Biotec, from now on referred to as Vectofusin) was added at a final concentration of 10 µg/ml to tissue culture-treated 96-well plates. For transductions of higher numbers of NK cells, 1 to 1.5 × 10^6^ NK cells were transduced on Retronectin-coated 12-well plates (1 ml final volume) or 3.5 to 4 × 10^6^ NK cells on Retronectin-coated 6-well plates (2.5 ml final volume) with 500 or 1,250 µl concentrated lentiviral particles, respectively, encoding CD19, CD33, CD123, or Cetux CARs. After 24 h, cells were supplemented with 2 ml (12-well) or 5 ml (6-well) fresh complete NK cell medium. Transduction efficiency was determined 3 to 4 days after transduction by flow cytometry, measuring BFP and/or CD34 (CAR) expression with phycoerythrin (PE)-conjugated QBend-10 CD34 antibody (Thermo Fisher Scientific).

### CAR NK Cell Enrichment

Three to four days after transduction, CAR NK cells were enriched on an OctoMACS™ Separator using MS columns or on a QuadroMACS™ Separator using LS columns after staining with CD34 microbeads (clone QBend-10) according to the manufacturer’s instructions (Miltenyi Biotec) and similarly as described previously for CAR T cells (28). In order to investigate the MACS enrichment efficiency, the CAR NK cell purity and yield, and the expression of the different CAR constructs on the transduced NK cells, the three cell fractions—before MACS, flowthrough, and after MACS—were collected and analyzed by flow cytometry for BFP expression and/or CD34 positivity. Before any functional analyses were performed, the MACS-enriched CAR NK cells were further cultured for at least an additional 2 days in a complete NK MACS medium.

### Blocking and Cytotoxicity Assay

To block the activating receptors, non-transduced and CAR NK cells were incubated with monoclonal REAfinity™ antibodies against NKG2D, DNAM-1, NKp30, NKp44, NKp46, and NKp80 (Miltenyi Biotec) at concentrations of 10 µg/ml in complete RPMI-1640 at 4°C. These REAfinity™ antibodies harbor mutations in their Fc receptors, which prevents their binding to human Fc receptors. Control cells were incubated in parallel with REAfinity™ isotype controls (Miltenyi Biotec). After the 2 h of incubation, the NK cells were added at 3:1, 1:1, and 0.3:1 ratios (without washing) to MOLM-14, NOMO-1, CMK, THP-1, REH and BV-173 cells seeded at 2 × 10^4^ cells/well in a 96-well U-bottom plate in complete RPMI-1640 medium. After 16 h, the cultures were harvested, and leukemic cell lysis was analyzed by flow cytometry, using propidium iodide (Sigma-Aldrich) for live/dead cell discrimination. The leukemic cells in the culture were specifically recognized by either EGFP expression (REH, MOLM-14) or after staining with CD15-FITC or CD33-FITC REAfinity™ antibodies. The samples were analyzed on a MACSQuant^®^ Analyzer 10 (Miltenyi Biotec). The specific lysis in % was calculated as [1 − number of viable target cells (sample)/number of viable target cells (control)] × 100%.

### NSG Xenotransplantation Mouse Model

All animal experiments were approved by the state animal research committee (LANUV, NRW, Germany). The use of leftover primary blasts from the initial AML diagnosis of children or adolescents treated within the AML-BFM study group was authorized by the ethics committee of the medical faculty at the University of Duisburg-Essen (application number 16-7069-BIO). Each AML-BFM trial was previously approved by their local institutional ethics committees: the Hannover Medical School for AML-BFM 2012 trial and registry (application number 13.03.12/La) and the University of Duisburg-Essen’s medical faculty for AML-BFM 2017 registry (application number 17-7462-BO). The primary blasts were injected into 8- to 16-week-old NOD.Cg-Prkdc^SCID^Il2rg^tm1Wjl^/SzJ (NSG) mice (Charles River Laboratories, Sulzfeld, Germany) and then sacrificed upon the clinical manifestation of the AML (Füchtjohann, Hanenberg, *in preparation*, 2022).

For analyzing the *in vivo* persistence of NK cells, 8- to 10-week-old NSG mice were treated by i.p. injection of 25 mg/kg of busulfan (Busilvex^®^, Busulfan, Medac, Darmstadt, Germany) and the next day intravenously transplanted with 10 × 10^6^ NK cells containing 50% transduced and 50% untransduced NK cells. Transduced NK cells co-expressed either soluble human IL15 and EGFP or human IL15 bound/tethered to the IL15 receptor α-chain (IL15-IL15R) and EGFP. Control mice were transplanted with 10 × 10^6^ untransduced NK cells. Each group consisted of 3 animals. Peripheral blood was analyzed at days 5, 10, 20, and 27 after lysis of erythrocytes with BD Pharm Lyse (BD Biosciences, San Jose, CA, USA) on a MACSQuant™ Analyzer 10 for murine CD18, human CD45, CD56, and EGFP-expressing cells (all antibodies were REA clones from Miltenyi Biotec) as described previously (28).

For the engraftment of REH cells expressing a firefly luciferase/EGFP fusion protein (REH^LucEG^) (28), 7- to 10-week-old NSG mice were treated by i.p. injection of 25 mg/kg of busulfan and the next day intravenously transplanted with 3.5 × 10^6^ REH^LucEG^ cells *via* tail vein injections. Two days later, the animals were randomized into different treatment groups and injected with 3.5 × 10^6^ NK cells containing 50% positive CD19 CAR NK cells co-expressing BFP, IL15, or IL15-IL15R and 50% untransduced NK cells (6 mice per group, 7 mice as untreated control). Luminescence analysis was performed on days 7, 15, 22, and 27 as previously described (20) to assess the progression/proliferation of the ALL blasts. Peripheral blood was obtained at days 8, 15, and 22 *via* the tail vein and, after lysis of erythrocytes with BD Pharm Lyse, analyzed on a MACSQuant™ Analyzer 10 for EGFP positive REH^LucEG^ cells and BFP, CD34, CD56, and CD94 NK cells (CD34-PE from Thermo Fisher Scientific, all other antibodies from Miltenyi Biotec).

### Fluorescence Microscopy

MACS-enriched CAR NK cells co-expressing BFP were stained with the QBend-10 CD34-PE antibody (as described above) and then co-cultured with EGFP-expressing REH cells in complete DMEM at an NK cell to target cell ratio of approximately 0.3:1. For these experiments, gelatin-treated 8-well slides with glass bottom (ibidi GmbH, Gräfelfing, Germany) were used. Bright-field and epifluorescence images were acquired at 37°C for 1–4 h after the seeding using an IX83 Inverted Microscope (Olympus, Hamburg, Germany), a 100× oil objective (NA 1.49, UAPON100XOTIRF, Olympus, Hamburg, Germany), and the cellSens Dimension software (Olympus, Hamburg, Germany). Images were processed using the open-source Fiji software (https://imagej.net/software/fiji/).

### Statistical Analysis

Statistical analyses were performed using GraphPad PRISM 9.0 using the log-rank test, one-way ANOVA with Tukey adjustment for multiple comparisons, and unpaired Student’s t-test for single comparison. p-Values ≤ 0.05 were considered significant and indicated with an asterisk.

## Results

To define optimal conditions required for the generation of CAR-expressing primary human NK cells and their expansion under Good Manufacturing Practice (GMP)-compliant conditions recently published in cooperation with Miltenyi Biotec (26), we systematically tested a number of variables that are known to influence the cytotoxicity of genetically modified NK cells.

### Lentiviral Pseudotypes for Efficient Transduction of Human NK Cells

Recently, two constructs derived from the envelope protein of the baboon endogenous virus, BaEV-Rless and BaEV-TR (14) have been published that are optimally suited for genetically modifying NK cells (21, 23). We additionally wanted to assess the transduction efficiency achieved in human NK cells with an alternative envelope pseudotype, GALV-TM, a chimera generated from the gibbon ape leukemia virus and the aMLV envelope proteins (34), that we previously used for the transduction of human CD34+ stem cells (35). For efficient virus production, our lentiviral vector plasmid pCL6EGNwo, expressing a fusion protein of EGFP and the neomycin resistance gene under the control of a modified SFFV promoter (27), was pseudotyped with the three envelopes. As additional controls, we also pseudotyped the lentiviral vector with the feline endogenous virus chimeric construct RD114-TR (31) and VSV-G (20).

**Figure 1:**
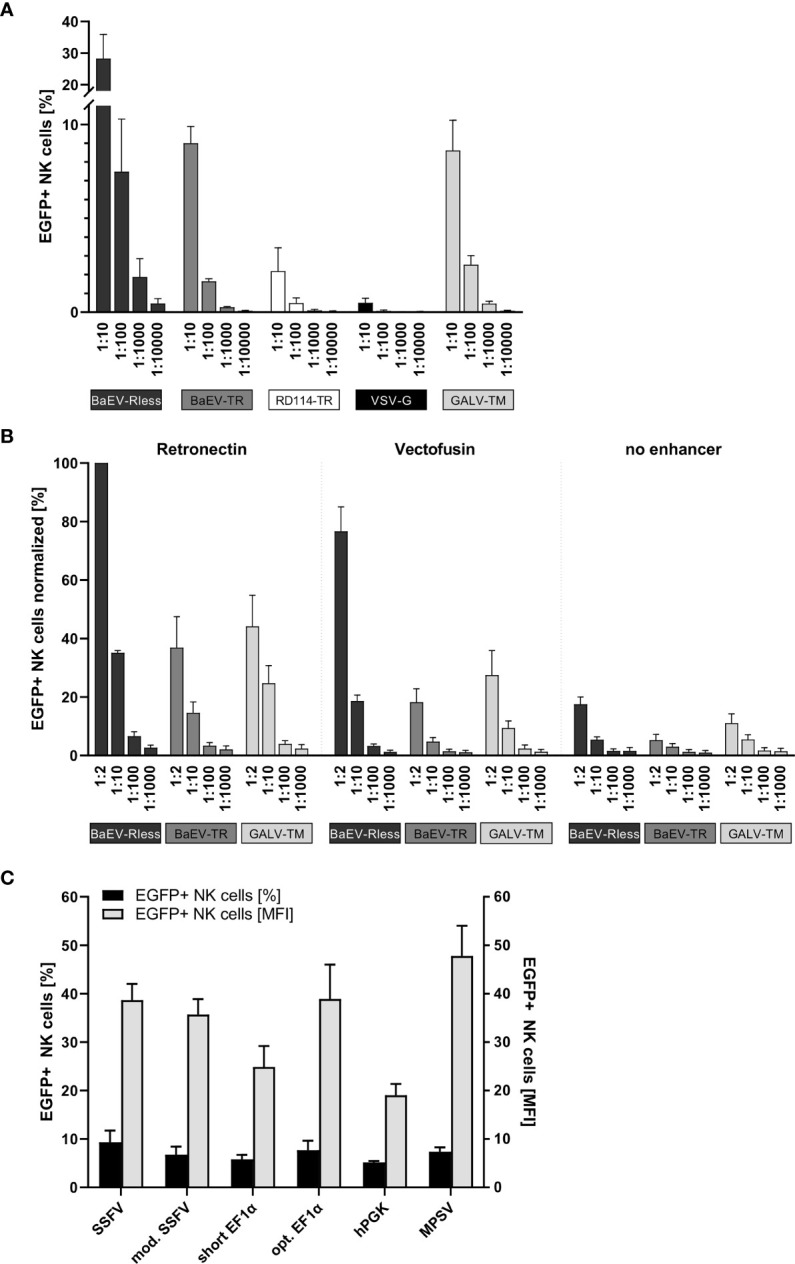
Systematic improvement of primary human NK cell transduction. Primary human NK cells were isolated and cultured as described in the body of the manuscript. **(A)** After 7 to 10 days of expansion, NK cells were transduced with serial dilutions of EGFP-expressing lentiviral particles pseudotyped with BaEV-Rless, BaEV-TR, RD114-TR, VSV-G, or GALV-TM. Three to four days after transduction, EGFP expression was analyzed by flow cytometry. Data are represented as mean ± SEM of four biological replicates. **(B)** Primary human NK cells were transduced with serial dilutions of EGFP-expressing lentiviral particles pseudotyped with BaEV-Rless, BaEV-TR, or GALV-TM, using Retronectin, Vectofusin, or no transduction enhancer, respectively. Three to four days after transduction, EGFP expression was analyzed by flow cytometry. Data are represented as mean ± SEM of five biological replicates. **(C)** Primary human NK cells were transduced with serial dilutions of lentiviral particles pseudotyped with BaEV-Rless, expressing EGFP under the control of the wild-type or modified SFFV, the wild-type or optimized EF1α, the hPGK, or the MPSV promoter. Three to four days after transduction, the expression of EGFP was analyzed. From the serial dilutions, samples with gene transfer rates of between 5% and 10% were analyzed for their EGFP expression intensity [mean fluorescence intensities (MFIs)]. Data are represented as mean ± SEM of four biological replicates.

Primary human NK cells were isolated and expanded as previously described (26) and then transduced with serially diluted (1:10 to 1:10,000) non-concentrated supernatants of freshly produced lentiviral vector particles on Retronectin-coated 96-well plates. Three to four days after transduction, NK cells were analyzed by flow cytometry for EGFP expression. As shown in **Figure 1A**, BaEV-Rless was the most efficient pseudotype, as 28% of NK cells were EGFP positive using 1:10-diluted viral supernatant; even a 1:1,000 dilution of this pseudotype was associated with a higher transduction efficiency than VSV-G at a 1:10 dilution. The lentiviral vectors pseudotyped with BaEV-TR or GALV-TM transduced approximately 9% (at 1:10) to 0.3% (at 1:1,000) of the NK cells. While being more efficient than VSV-G, the RD114-TR pseudotype was still rather inefficient with gene transfer rates ranging from 2.2% to 0.1%.

### Retronectin *vs.* Vectofusin for Enhancing the Transduction of Human NK Cells

To promote the binding of lentiviral particles on the surface of target cells and thereby increase the likelihood of viral entry, cationic culture additives such as polybrene or protamine sulfate have been used during the transduction procedure and neutralize the electrostatic repulsion between the opposing negatively charged membrane bilayers (36, 37). Vectofusin is such a histidine-rich cationic amphipathic peptide that was developed to promote efficient lentiviral transduction of primary human cells (38), including T and NK cells (39). Alternatively, a recombinant chimeric fragment derived from human fibronectin, CH-296 or Retronectin, is used since 1996 in research settings and in clinical trials to promote colocalization of viral particles and mammalian target cells, thereby increasing the efficiency of the genetic modification for both adherent and non-adherent cells (32, 33). Although the effects of Vectofusin and Retronectin on the transduction of different non-adherent cell types including human CD34+ hematopoietic stem cells (38) and also primary human NK cells (23, 39) have been reported, this type of comparison has not been performed for the lentiviral BaEV pseudotype that mediates the most efficient gene transfer into primary human NK cells (**Figure 1A**).

Therefore, we transduced primary human NK cells with the pCL6EGNwo vector (27) pseudotyped with BaEV-Rless, BaEV-TR, or GALV-TM using serial dilutions, ranging from 1:2 to 1:1,000. Transduction was performed in flat-bottom 96-well plates, either coated with Retronectin or after supplementing Vectofusin to the culture medium. Control NK cells were transduced in 96-well cell culture plates without any additional transduction enhancer. The gene transfer efficiencies into the human NK cells were analyzed 3 to 4 days later by flow cytometry, and the values were normalized by assigning 100% to the highest transduction efficiency achieved within the cell series of each donor. Remarkably, the BaEV-Rless pseudotype on Retronectin mediated the highest gene transfer in each of the four donors and was therefore set at 100%, while the other two pseudotypes BaEV-TR and GALV-TM did not even achieve 50% of the BaEV-Rless transduction efficiency (**Figure 1B**). Using Vectofusin with the BaEV-Rless-pseudotyped lentiviral particles resulted in roughly 80% of the Retronectin-assisted gene delivery and was clearly better than the gene transfer efficiencies observed with the other two pseudotypes BaEV-TR and GALV-TM. The gene transfer without any transduction enhancer was very low regardless of the glycoprotein used. Therefore, for the remaining experiments in this study, we chose to perform the lentiviral transductions of primary human NK cells with the BaEV-Rless pseudotype on Retronectin-coated plates.

### A Promoter for High-Level Transgene Expression in Human NK Cells

We have previously shown that the U3 region of the MPSV as an internal promoter in our self-inactivating (SIN) lentiviral vectors is the best choice for achieving high-level gene expression in primary human T cells (27). Here, in order to identify an optimal internal promoter for stable high-level CAR expression in primary human NK cells, we tested the MPSV and two additional viral promoters as internal promoters in our lentiviral pCL6EGNwo vector (27): the U3 regions from SFFV (40) with one (mod. SFFV) or with two enhancer regions (SFFV). We also included two promoters of human cellular housekeeping genes, namely, the human phosphoglycerate kinase-1 (hPGK) (27) and the human elongation factor 1α (EF1α) promoter with (opt. EF1α) or without (short EF1α) an optimized splicing unit as internal promoters.

Primary human NK cells were transduced with serially diluted BaEV-Rless-pseudotyped lentiviral vectors expressing the EGFP-neomycin fusion as marker gene under the control of the different promoters. Three to four days after transduction, NK cells were analyzed for EGFP expression by flow cytometry. We compared the mean fluorescence intensity (MFI) of the different promoters in samples with a gene transfer of approximately 5%–10%, as cells with this gene transfer rate most likely carry only one copy of the vector integrated into their genome (27), thereby eliminating the bias of multiple integrations on the transgene expression levels. As shown in **Figure 1C**, the hPGK and short EF1α promoters with MFIs of 19.0 and 24.9, respectively, were associated with the lowest transgene expression levels. Optimizing the splice unit of EF1α by shortening the intron and correcting an open reading frame in the intron improved the transgene expression levels of the optimized EF1α promoter to those of the SFFV promoters with MFIs ranging from 35.7 to 38.9. However, the MPSV U3 promoter with an MFI of 47.8 still provided the highest level of transgene expression, and thus it was chosen for the expression of CAR constructs in primary human NK cells for our subsequent experiments.

### Transduction and Enrichment of Human CAR NK Cells

**Figure 2:**
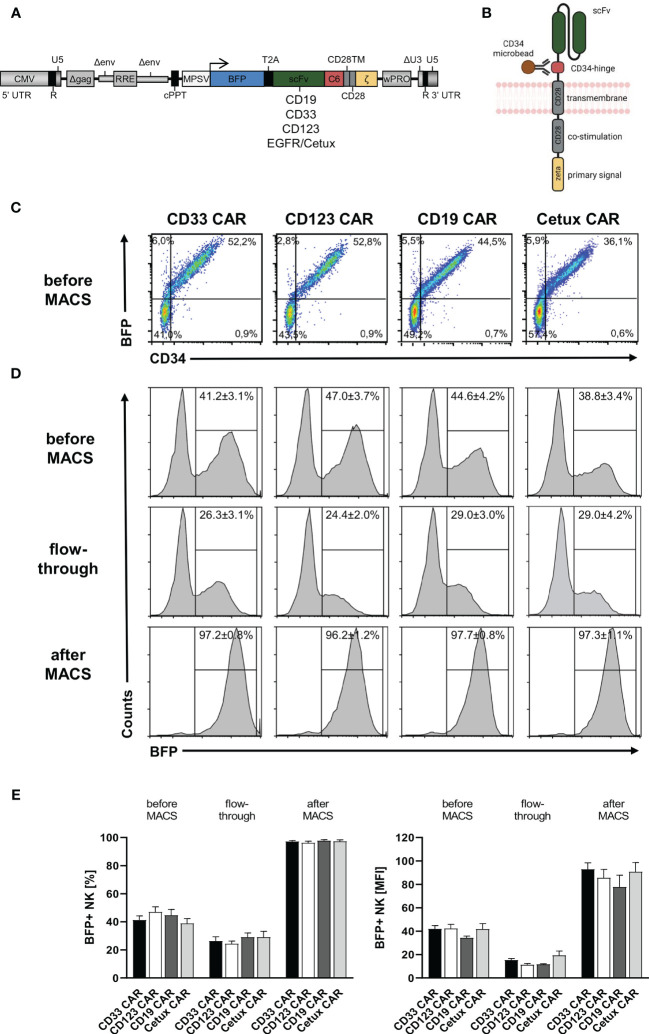
CD34-hinged CAR expression and enrichment in primary human NK cells. Primary human NK cells were transduced with the lentiviral vectors delineated in panels **(A, B)** encoding BFP *in cis* with CD34-hinged CD33, CD123, CD19, or Cetux CARs. **(B)** Schematic representation of CAR construct including CD34-derived C6 hinge for microbead interactions. **(C)** Three to four days after lentiviral CAR transduction, NK cells were analyzed for BFP and CAR (via CD34-PE staining) expression by flow cytometry. Representative dot plots are shown. **(D, E)** CD34-hinged CAR NK cells were enriched *via* magnetic cell sorting using CD34 microbeads and three fractions (before MACS, flowthrough, and after MACS) were analyzed for the content of NK cells expressing BFP by flow cytometry. The histogram blots show representative data. Data are represented as mean ± SEM of at least four biological replicates.

After the establishment of an optimized transduction protocol, primary human NK cells with low CD33 expression were transduced with 5-fold concentrated BaEV-Rless-pseudotyped lentiviral particles encoding BFP *in cis* with CD19, CD33, CD123, or EGFR CARs, harboring our CD34-derived hinge (20, 29), on Retronectin (**Figures 2A, B**). Three to four days after transduction, the expression of the CARs was analyzed after staining of the C6 hinge region with the QBend-10 antibody and detecting BFP expression by flow cytometry. As shown in **Figure 2C**, CD34/CAR expression strongly correlated with BFP expression for all four CAR constructs. To purify the CAR NK cells, we then performed MACS enrichment on an OctoMACS™ separator using MS columns and CD34 microbeads binding to the C6 hinge. As an indicator of the content of transduced NK cells, BFP expression was analyzed in three fractions: before MACS, the flowthrough, and after MACS (representative samples in **Figure 2D**). Before MACS enrichment, we obtained on average of between 38.8% ± 3.4% (Cetux) and 47.0% ± 3.7% (CD123) CAR-positive NK cells. Enrichment of CAR NK cells on MACS columns led to purification rates of between 96.2% ± 1.2% (CD123) and 97.7% ± 0.8% (CD19). However, the flowthrough contained relatively high percentages of CAR NK cells (between 24.4% ± 2.2% for CD123 and 29.0% ± 4.2% for Cetux), albeit with much lower MFIs than the CAR NK cells before or after MACS (**Figure 2E**).

### Reduction of Background Killing of Leukemic Cell Lines by Activated Human NK Cells

For more than 40 years, it is well established that after stimulation, primary human NK cells can exhibit high cytotoxic activities against human leukemic cells (41). This cytotoxicity is often mediated by the expression of antigens on the leukemic blasts that are ligands for activating receptors on NK cells (42). As *in vitro* expanded NK cells are highly activated (26), they often recognize and thus kill leukemia cells independently of CAR antigen binding (42). In order to eliminate this “background” killing for follow-up assays, we planned to systematically block activating receptors of the NK cells in cytotoxic experimental settings. To this end, we co-cultured cells of four AML cell lines and two B-cell precursor ALL cell lines overnight with three ratios of PB-derived 7- to 14-day-old NK cells with the effector-to-target cell ratios of 3:1, 1:1, and 0.3:1. In parallel, overnight co-cultures were set up with the same NK cells, albeit preincubated with monoclonal antibodies that recognized and block either three (NKG2D, NKp30, and NKp46) or six (NKG2D, DNAM-1, NKp30, NKp44, NKp46, and NKp80) activating receptors on NK cells (11, 43). Although these recombinant antibodies were all mutated in their Fc domains and therefore should not bind to human Fc receptors, we still included a co-culture condition with NK cells that were preincubated with the appropriate isotype controls.

**Figure 3:**
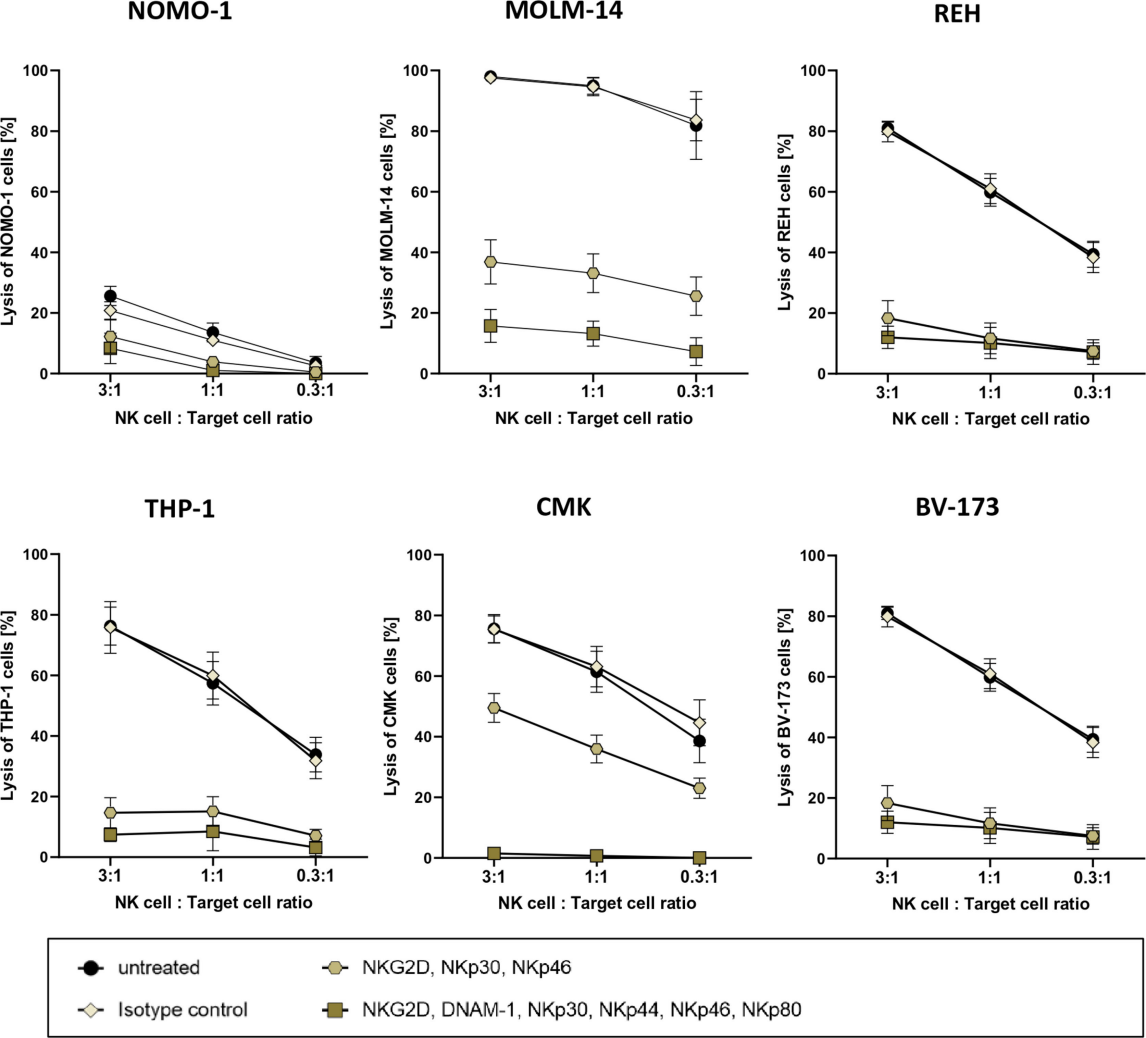
Cytotoxic potency of activated primary human NK cells against acute leukemia. Killing of various leukemic cell lines by activated primary human NK cells was analyzed by co-culture of NK cells and target cells at several ratios. Activated primary human NK cells were incubated with blocking antibodies against activation receptors (as stated) and co-cultured with NOMO-1, MOLM-14, REH, THP-1, CMK, and BV-173 cells at various ratios to determine cytotoxic activity of NK cells. After 16 h of co-incubation at 37°C, the cytotoxicity of the treated and untreated NK cells was analyzed *via* flow cytometry, and the degree of the cell lysis was analyzed as described in the *Material and Methods*. The graphs represent mean ± SEM of four biological replicates.

As demonstrated in **Figure 3**, the degree of target cell lysis by activated NK cells seemed to be cell line-specific. On the other hand, three AML cell lines [MOLM-14 (AML-M5), THP-1 (AML-M4), and CMK (AML-M7)] and two B-cell precursor leukemic lines [REH and BV-173] were killed efficaciously by the activated NK cells in the absence of blocking antibodies and also in the presence of isotype controls; NOMO-1 (AML-M5) cells were resistant to NK cell killing. Importantly, preincubation with all six antibodies ameliorated the cytotoxicity of the NK cells against the five susceptible cell lines quite efficiently; however, using the three antibodies against NKG2D, NKp30, and NKp46 only partially reduced the NK cell-mediated killing for two AML cell lines, MOLM-14 and CMK (**Figure 3**).

### CAR NK Cells Effectively Kill AML and ALL Cell Lines as Well as Primary AML Blasts *In Vitro*

**Figure 4:**
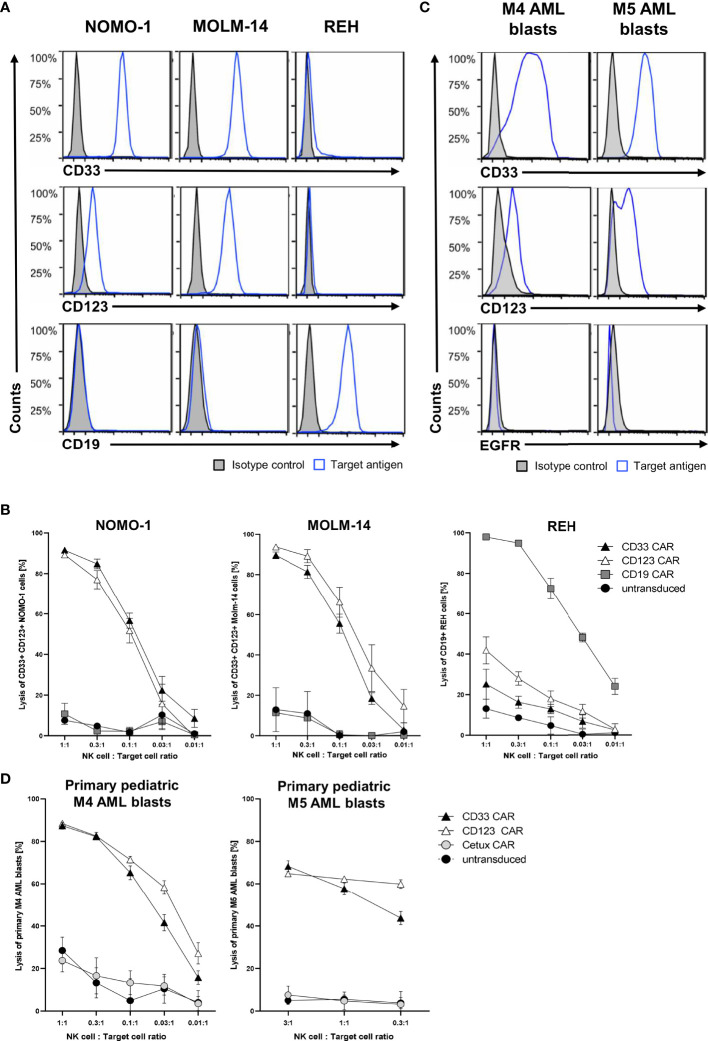
Cytotoxic activity of CAR NK cells against leukemic cells. **(A)** AML cells NOMO-1 and MOLM-14, and ALL cells REH were analyzed for their surface expression of CD19, CD33, and CD123. **(C)** Primary pediatric M4 and M5 AML blasts were analyzed for their surface expression of CD33, CD123, and EGFR using conjugated antibodies by flow cytometry. CAR-transduced and microbead-enriched primary human NK cells were blocked as described previously and co-cultured with NOMO-1, MOLM-14, and REH cells **(B)** or primary pediatric M4 and M5 AML blasts **(D)** at various effector-to-target cell ratios to determine the specific cytotoxic activity of CAR NK cells. The graphs represent mean ± SEM of at least five (cell lines) or three (AML blasts) biological replicates.

After successfully establishing a blocking protocol, we specifically tested the functionality of CAR NK cells against one ALL and two AML cell lines. To this end, MACS-enriched CAR NK cells of at least five healthy donors with low CD33 expression (data not shown) were blocked with all six monoclonal antibodies described above and then co-cultured overnight with NOMO-1, MOLM-14 (both CD19− CD33+ CD123+), and REH (CD19+ CD33− CD123-) cells at various effector-to-target cell ratios. Flow cytometric analysis revealed the expression patterns of CD19, CD33, and CD123 for each of the target cell lines (**Figure 4A**). After 16 h of co-culture, CD33 and CD123 CAR NK cells efficaciously killed NOMO-1 and MOLM-14 cells with approximately 90% specific lysis at an effector-to-target cell ratio of 1:1 (**Figure 4B**). Interestingly, although CD123 is expressed much lower on NOMO-1 cells than CD33 (**Figure 4A**), CD123 CAR NK cells performed as well as CD33 CAR NK cells in the NOMO-1 co-cultures. REH cells were highly efficaciously (>95% at 1:1) eradicated by CD19 CAR NK cells, but not by the other CAR constructs (**Figure 4B**). Importantly, the lysis was strongly dependent on the specific antigen expression, and we did not observe off-target toxicities in these experiments.

Next, we hypothesized that the CAR NK cells would also specifically kill primary AML blasts obtained at diagnosis and expanded in NSG mice (Füchtjohann, Hanenberg, *manuscript in preparation 2022*). As AML blasts can express low levels of CD19, we used the EGFR-recognizing Cetux CAR NK cells as negative controls. Flow cytometric analysis revealed the expression patterns of EGFR, CD33, and CD123 for the primary AML blasts used (**Figure 4C**). The results in **Figure 4D** demonstrated that the CD33+ CD123+ primary AML-M4 blasts were specifically killed (up to 90% at 1:1), while only 60% of CD33+ CD123+ primary AML-M5 blasts were eliminated by both CD33 and CD123 CAR NK cells at 3:1 and 1:1 ratios, respectively.

**Figure 5:**
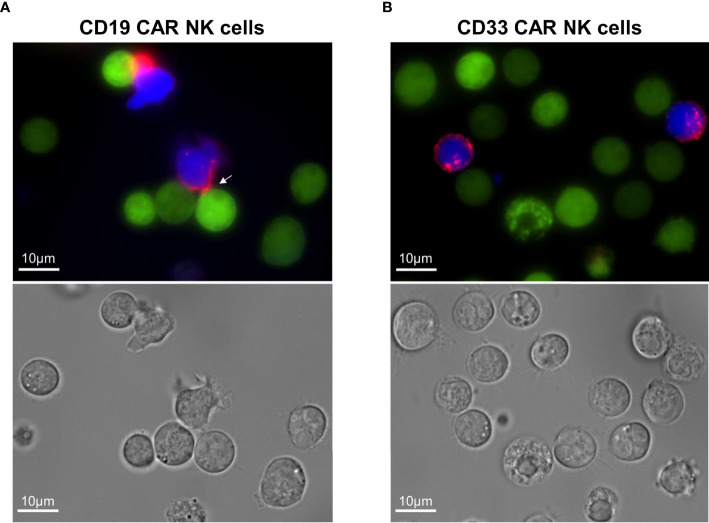
Visualization of the immunological synapses formed between CD19 CAR NK cells and CD19+ REH cells. **(A)** The immunological synapse between CD19 CAR NK cells and CD19+ REH cells was analyzed by fluorescence microscopy, visualizing the CAR expression (CD34-PE-conjugated antibody, red) on MACS-enriched CAR NK cells (BFP expression, blue) in relation to REH cells (EGFP expression, green). **(B)** MACS-enriched CD33 CAR NK cells served as negative controls. Contrast settings are uniform for both images for the channels: brightfield, 377/447 and 470/525 nm. The contrast in channel 545/620 nm was adjusted to maximize the visibility of the cell-to-cell contacts.

Finally, we took advantage of the fact that our CARs contained the CD34-derived C6 hinge to visualize the specific interaction of CD19 CAR NK cells with the CD19-positive REH cells in the presence of blocking antibodies. For the fluorescence microscopy analysis, the PE-conjugated QBend-10 antibody was employed to label the hinge region of CD19 or CD33 CAR NK cells, which both co-expressed BFP, while the target REH cells expressed EGFP. As indicated by the white arrow in **Figure 5A**, the CD19 CAR constructs on the transduced NK cells accumulated in immunological synapses formed between 1 and 4 h between NK and REH cells. In contrast, the CD33 CAR constructs were equally dispersed on the cell surfaces of CD33 CAR NK cells (**Figure 5B**), suggesting that no specific interaction with the CD33-negative REH cells occurred.

### CD19 CAR NK Cells Effectively Kill CD19+ ALL Cells *In Vivo*

**Figure 6:**
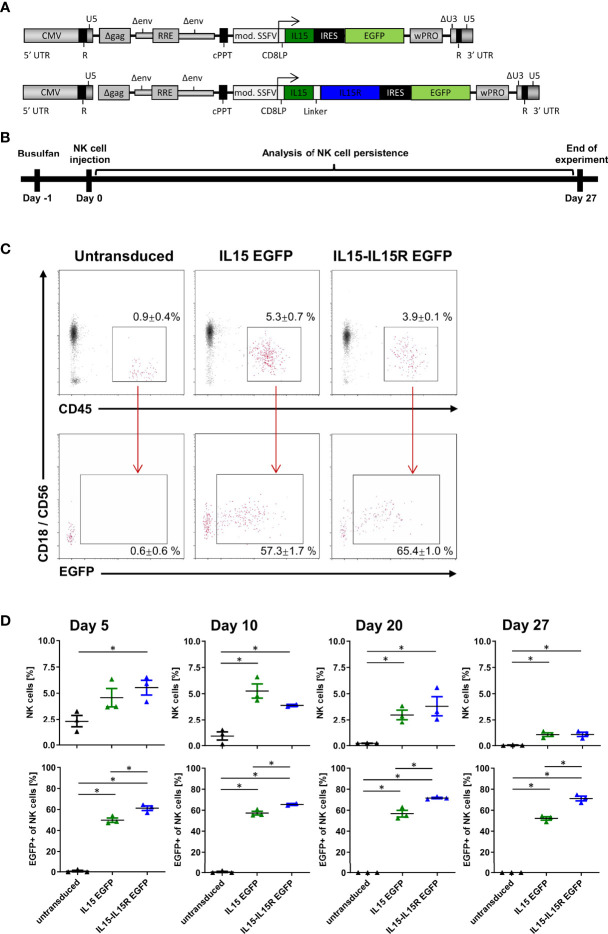
NK cells rely on endogenous IL15 production for *in vivo* persistence in NSG mice. **(A)** Schematic representation of the vector maps of the IL15 constructs used for NK cell transduction. **(B)** Primary human NK cell preparations consisting of 50% EGFP/IL15 or EGFP/IL15-IL15R and 50% untransduced NK cells (or 100% untransduced cells as control group) were injected into busulfan-preconditioned mice (3 animals per group). On days 5, 10, 20, and 27, NK cell persistence in the bloodstream was analyzed by mouse CD18, human CD45, and CD56 staining as well as EGFP expression. **(C)** Example of gating strategy showing representative data from day 10. Values indicate mean ± SEM of all three animals. **(D)** NK cell persistence in the blood including ratios of transduced to untransduced NK cells. Data are represented as mean ± SEM. Significances of p<0.05 are indicated by an asterisk (*).

For *in vivo* application of activated NK cells in humans, it is paramount that NK cells do not possess autocrine stimulation loops that would ensure their survival beyond a few days in patients (44, 45). Therefore, allogeneic NK cell therapies are usually accompanied by the subcutaneous application of IL2 or intravenous infusions of lentiviral IL15; both treatments are actually associated with severe side effects in patients (44, 45). In order to evaluate the impact of IL15 signaling on the persistence of NK cells *in vivo*, we generated two lentiviral IL15 vectors (**Figure 6A**): the first expressed soluble human IL15 and EGFP, and the second human IL15 tethered to the IL15 receptor α-chain (IL15-IL15R) and EGFP. Importantly, both IL15 constructs were able to mediate the expansion of primary NK cells in *in vitro* experiments in the absence of any other growth factors (data not shown). Next, NSG mice were injected with 5 × 10^6^ EGFP+ NK cells expressing either soluble IL15 or IL15-IL15R and 5 × 10^6^ untransduced NK cells. We used this 1:1 ratio *in vivo* to investigate potential survival advantages of IL15 expressing NK cells over untransduced NK cells in each mouse and also to understand the impact of IL15-tethering for both populations. The persistence of human NK cells was assessed for 27 days (**Figure 6B**), using the gating strategy shown in **Figure 6C** for the peripheral blood analysis on day 10. **Figure 6D** reveals that the NK cell survival was highly dependent on IL15 signaling, as the percentages of NK cells in the blood of the mice transplanted with untransduced NK cells progressively decreased over time. In contrast, both strategies to express IL15 *in vivo* drastically improved the initial NK cell engraftment and also the persistence of NK cells at all sampling time points. Remarkably, the ratio of EGFP positive to negative cells stayed rather constant at 1:1 for the soluble IL15, while the IL15-IL15R expressing NK cells appeared to slowly outgrow the untransduced cells over time: after 27 days, approximately 70% of all NK cells in the blood of the animals were EGFP positive.

**Figure 7:**
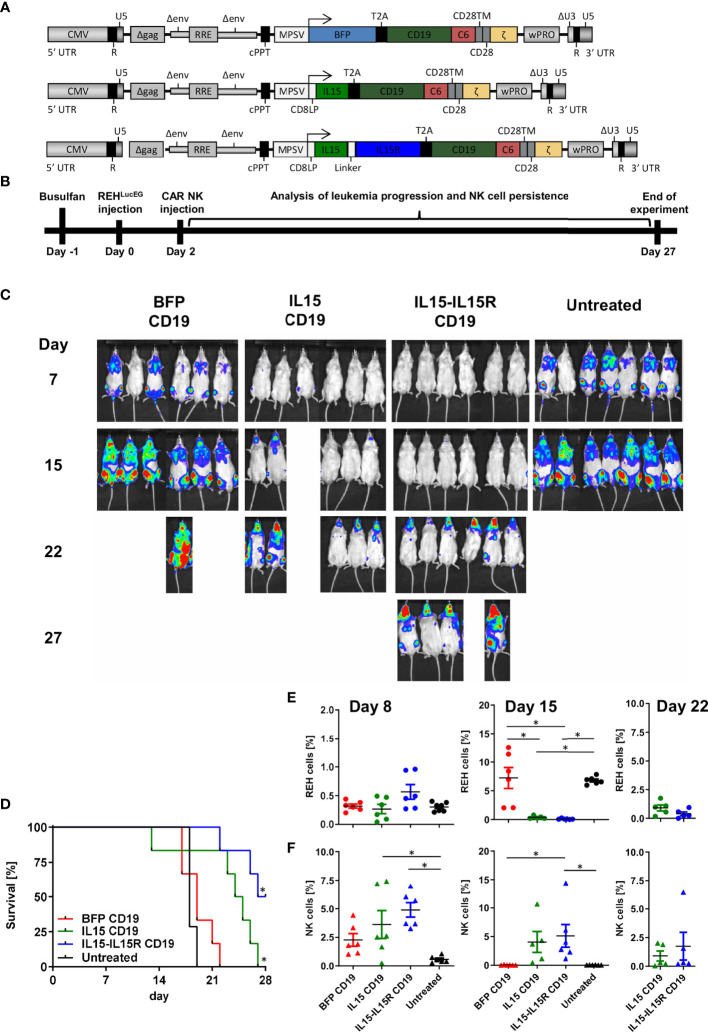
CAR NK cells rely on endogenous IL15 production for leukemia control in an ALL xenograft model. **(A)** Schematic representation of vector maps of CAR constructs used for NK cell transduction. **(B)** Primary human NK cell preparations consisting of 50% BFP/CD19 CAR, IL15/CD19 CAR, or IL15-IL15R/CD19 CAR and 50% non-transduced NK cells were injected 2 days after xenotransplantation of REH^LucEG^ into busulfan-preconditioned NSG mice (6 animals per group, 7 animals as untreated control). **(C)** On days 7, 15, 22, and 27, leukemia progression was analyzed by luminescence imaging in a Caliper device. **(D)** Kaplan–Meier survival curves for the three treatment groups and the untreated control animals. **(E)** REH^LucEG^ and **(F)** NK cell persistence in the bloodstream was analyzed by CD19, CD34, CD45, and CD56 staining as well as EGFP expression by flow cytometry. Data are represented as mean ± SEM. Significances of p<0.05 are indicated by an asterisk (*).

Finally, we used an ALL xenograft model in NSG mice to evaluate the impact of IL15 signaling for the survival and leukemia control of CAR NK cells *in vivo*. For this experiment, three CD19 CAR vectors were generated (**Figure 7A**) co-expressing *via* a T2A site either BFP, soluble human IL15 (17), or human IL15 tethered to the IL15 receptor α-chain (IL15-IL15R) (46). For the *in vivo* experiments, NSG mice were transplanted intravenously with 3.5 × 10^6^ REH cells expressing a firefly luciferase/EGFP fusion protein (REH^LucEG^). Two days later, 3.5 × 10^6^ total primary human NK cells with 50% non-transduced and 50% transduced cells were injected *via* the tail vein (**Figure 7B**). Untreated NSG mice showed rapid leukemia progression and had to be sacrificed between days 17 and 19 (**Figures 7C, D**). Surprisingly, treatment of the mice by infusion of BFP/CD19 CAR NK cells did not improve the survival at all (**Figures 7C, D**). For these two groups, the REH cells made up between 0.3% and 0.4% of peripheral blood cells on day 7 and between 3.3% and 7% on day 15 (**Figure 7E**). Interestingly, the BFP/CD19 CAR NK cells were still detectable at day 8 (2.3% of peripheral blood cells) but did not persist in the blood beyond this time point (**Figure 7F**). When the CD19 CAR NK cells additionally expressed IL15 or IL15-IL15R instead of BFP, the leukemia progression was markedly reduced as seen by luminescence imaging (**Figure 7C**) and peripheral blood analysis (**Figure 7E**). We attributed the improved survival of the animals to the robust increase in the persistence of CAR NK cells, as these immune effector cells were detected at high levels on day 15 (4% for IL15/CD19 and 5.1% for IL15-IL15R/CD19 CAR NK cells) and were even present at lower levels on day 22 in the peripheral blood of the animals (**Figure 7F**). Importantly, however, the animals in the IL15 and IL15-IL15R groups all showed ALL persistence in central nervous system (CNS) lesions (**Figure 7C**). These lesions partly led to neurological deficits/abnormalities, and the majority of these animals had to be sacrificed between days 22 and 27. At the termination of the experiment, three NSG mice of the IL15-IL15R/CD19 CAR NK cell group were still alive, albeit all affected by CNS leukemia.

## Discussion

The application of risk-adapted treatment protocols has greatly improved the survival of children and adolescents with ALL and acute myeloid leukemia (AML) during the past four decades (47). However, a significant number of patients, at least 10% of pediatric ALL and 25% of pediatric AML, still suffer from relapse and/or refractory disease and despite all efforts ultimately succumb to their leukemia (48–51). For these patients, phase I/II clinical trials with adoptive cellular therapies generally were only beneficial in post-transplantation settings, where lymphocyte infusions of the stem cell donors are often sufficient to treat minimal residual disease and even full-blown relapses (52). Infusions of allogeneic NK cells have also been tested in clinical trials and were tolerated well without any GvHD; however, the therapeutic benefits were rather limited, and the concomitant treatment with IL2 or IL15 was associated with profound acute toxicities (11). Recently, antibody-based immunotherapies with bispecific T cell engagers or CAR T cells have achieved remarkable initial success, predominantly for B cell-associated malignancies (53, 54). However, for patients with relapsed/refractory leukemias, the generation of autologous CAR T cells often takes too much time, is technically quite difficult in small children or heavily pretreated patients, and rather impossible for AML patients due to the lack of suitable target antigens for the long-term persistent CAR T cells (53, 54). Here, generating allogeneic CAR NK cells for *off-the-shelf* usage and with limited persistence *in vivo* in patients might be ideal for inclusion in salvage treatment protocols for relapsed/refractory patients, e.g., as a blast-reductive treatment/bridge to allogeneic stem cell transplantation. Taking advantage of the new possibilities to generate allogeneic CAR NK cells on the CliniMACS Prodigy^®^ platform (Miltenyi Biotec) with chemically defined media, we systematically tested variables affecting the transduction and cytotoxic efficacy of allogeneic NK cells with these tools.

The efficacy of CAR immune effector cells to eliminate their target cells is influenced by several factors, including the affinity of the scFv present in the CAR construct, the expression levels of the CAR itself on the effector cells, and availability of the targeted antigen/epitope on the target cell (8, 29). Thus, for CARs with a low affinity or when the target antigen is expressed at low levels, high and stable CAR expression is crucial to ensure excellent antitumor cytotoxicity. To this end, the internal promoter in the SIN lentiviral vector and the CAR/transgene sequence(s), e.g., after the codon optimization, are the main tools to improve CAR expression levels. As we already employed cDNAs optimized for human codon usage, the systematic testing of various promoters was our final step to optimize CAR expression in primary human NK cells. To our knowledge, only Allan et al. tested multiple internal promoters in lentiviral vectors for the transduction of human primary NK cells and finally favored the short human EF1α promoter as the best out of eight candidates for their bicistronic CAR expression cassette (55). In our study, we observed the same relationships when comparing the transgene expression levels of the short EF1α, the EF1α with optimized splicing cassette, and the hPGK promoters, but all eight promoters from Allan et al. still appear to be much weaker than the MPSV promoter that we originally established for robust lentiviral CAR expression in human T cells (27). The original EF1α (56), the human CMV (14), or the SFFV promoter (23, 39) was also used in other studies to express CAR constructs in primary human NK cells. However, since NK cell culture and transduction protocols differ greatly between groups, we analyzed the activity of these commonly used promoters in NK cells expanded in the NK MACS medium from Miltenyi and realized that the MPSV promoter outperformed the hPGK, both SSFV and both EF1α promoters, which is in line with our CAR T cell study (27).

A major advantage of HIV-1-derived lentiviral vectors is their promiscuity in accepting multiple heterologous viral envelopes as pseudotypes, thus allowing a wide tropism of target cells (57). While T cells can readily be genetically modified with the VSV-G pseudotype, neither quiescent nor activated NK cells express sufficient levels of the low-density lipoprotein receptors that are employed by VSV-G pseudotypes to enter target cells (23, 56). Changing the lentiviral pseudotype to BaEV-TR (23, 56) or RD114-TR (39) has resulted in better transduction efficiencies, as the cellular glycoproteins that serve as viral receptors, ASCT-1 and/or ASCT-2, are higher expressed on activated but not on naïve/resting NK cells (23, 56). Noteworthy here is that IL2 priming of human NK cells especially leads to upregulation of the glutamine transporter ASCT2 (14, 58). We also showed that GALV-TM-pseudotyped lentiviral vectors facilitate robust transduction of primary human NK cells, probably as the viral receptor PIT1 is sufficiently expressed on activated human NK cells (59, 60). The three envelopes BaEV-TR (14), RD114-TR (31), and GALV-TM (34) are actually chimeric/fusion proteins between the surface units of the three viral envelopes and the cytoplasmic domains of the aMLV; this is necessary as the lentiviral protease from HIV-1 cannot cleave the R-peptide of the three wild-type glycoproteins but can process the one of aMLV during the extracellular maturation process of the budding lentiviral particles (14, 31, 34). In the BaEV-Rless glycoprotein, the R-peptide is already removed from the wild-type envelope, which resulted in a drastically enhanced fusogenic capacity of this pseudotype already during virus production in the HEK293T cells, as described by Girard-Gagnepain et al. and others (14, 61). Nevertheless, despite the huge syncytia formation, the release of physical particles seems to be almost similar for both pseudotypes, BaEV-Rless and BaEV-TR (14). In agreement with this work, our human codon-optimized BaEV-Rless version also here outperformed all other envelopes and provided by far the most efficient gene transfer into primary human NK cells.

In our studies, the use of the transduction enhancer Retronectin was associated with an 8-fold increase in gene transfer efficiency over the control wells without any enhancer and thus superior to Vectofusin, which mediated a 7-fold increase. For research purposes, these values are quite comparable; however, for clinical applications, Miltenyi has adapted the use of their Vectofusin reagent for the automated processes in the closed tube system on the CliniMACS Prodigy^®^ (26). Thus, using Vectofusin will thus be the easiest way to achieve high transduction efficiencies for clinical CAR NK cell products.

We previously established a novel element for CARs, the CD34-derived C6 hinge, that facilitates detection and enrichment of CAR T cells in clinical processes and does not negatively influence the functional characteristics of genetically transduced and enriched T cells *in vitro* and *in vivo* in mice (28). In the present study, we demonstrated that C6 as a hinge in CAR constructs expressed on human NK cells also allows to easily detect transduced NK cells *in vitro* or the peripheral blood of mice using the QBend-10 antibody by flow cytometry. Additionally, the C6 hinge also facilitates rapid enrichment of genetically modified CAR NK cells to purities of >95% using CD34 microbeads on MACS columns. However, compared to CAR T cells (28), enriching CAR NK cells with the same CD19 CAR construct with the C6 hinge resulted in a higher loss of CAR NK cells in the flowthrough, 29.0% for CAR NK cells (**Figure 2**) compared to only 11.7% for CAR T cells (28). We believe that these differences in the MACS enrichment efficiency are due to the lower expression levels (MFIs) of the CAR constructs on NK cells (data not shown). Even when the CAR T and NK cells originate from the same donors and both cell types showed similar gene transfer rates, more CAR NK cells are lost in the flowthrough (data not shown). One possible explanation is that the MPSV promoter, although still the best promoter tested for NK cells, might not express transgenes in NK cells as strongly as it does in T cells. However, in clinical settings, the loss of transduced NK cells in the flowthrough might not be as relevant as for autologous CAR T cell therapy, as the NK cell numbers can easily be adapted in allogeneic settings and one CAR NK cell product from apheresis might still be sufficient for several recipients (26). The key point here is that allogeneic NK cells with high CAR expression levels can readily be purified on a system that is compatible with GMP conditions and where the CD34 microbeads might not need to be removed (62, 63).

In order to verify the specific functionality and cytotoxic activity of our enriched CAR NK cells, we overcame the impediment of profound NK cell killing of leukemic cells by introducing blocking antibodies against six activating NK cell receptors in the co-cultures. Previously, a decrease in the NK cell cytotoxicity was reported when singularly blocking DNAM-1 (64) or NKG2D (65) on NK cells prior to incubation with AML cells. Additionally, Boermann and colleagues tested the effects of blocking NKG2D, DNAM-1, NKp30, NKp44, and NKp46 on primary human NK cells prior to co-culture with rhabdomyosarcoma cell lines (66). Others just overexpressed the target antigen for their CAR NK cells on cells that were not attacked per se by activated NK cells (26). Currently, we are in the process of systematically analyzing the effect(s) of each ligand on leukemic blasts for NKG2D, DNAM-1, NKp30, NKp44, NKp46, and/or NKp80 on *in vitro* activated and expanded NK cells (data not shown). So far, the success of blocking individual receptors seems to be target cell line-dependent. Paramount is that despite the blocking of their activating receptors, the MACS-enriched NK cells expressing CD19, CD33, or CD123 CARs still exhibit highly specific cytotoxic activity against ALL and AML cells *in vitro*. Fluorescence microscopy of CD19 CAR NK cells attacking CD19-positive REH target cells revealed that the C6 hinge is also an ideal tool to visualize the immunological synapses where the direct interaction between the CAR NK cells and the target cell occurs. We additionally demonstrated that primary AML blasts from pediatric patients expanded in NSG mice can be used as important target cells to determine the specific cytotoxicity of allogeneic CAR NK cells against these primary blasts.

Although CD19 CAR NK cells efficaciously killed the two CD19-positive B-cell precursor ALL cell lines REH and BV-173 in overnight cultures *in vitro*, our *in vivo* experiments clearly demonstrated that support of the CD19 CAR NK cells by IL15 co-expression is an absolute prerequisite for the control of the ALL blasts *in vivo.* While the co-expression of soluble IL15 was required to increase survival of the animals, as also reported by others (17, 67), the group of animals that received CAR NK cells, where IL15 is tethered to the IL15 receptor α-chain, lived slightly longer. These results seemed to confirm previous observations by Imamura et al. (68), where the tethered IL15 sustained NK cell survival and expansion in an autocrine stimulation and might mediate survival and growth advantages over NK cells co-expressing non-membrane-bound IL15. However, the initial accumulation of REH cells in the CNS already at day 15 and then strongly at day 22 suggested that migration and/or defect for NK cells with soluble IL15 expression might exist for the CNS, while human CAR NK cells with IL15 tethered to the IL15R might be better suited to survive and function in the CNS of mice. Thus, the survival of the REH leukemic blasts in the CNS of the animals might simply reflect the reduced capabilities of NK cells to cross the blood–brain barrier of the animals. Noteworthy here is that we used the same REH ALL mouse model in our previous publication (28) where we showed that the C6 hinge in a CD19 CAR is as efficacious as a commonly used CD8-derived hinge to control B-cell leukemia *in vivo* by CAR T cells. In this work, all mice in the CD19 CAR T cell groups survived, and no REH leukemia cells were detected in the CNS of the animals at any time point (28). Whether human CAR NK cells have defects in the homing to the CNS only in NSG mice or whether diminished migration of human CAR NK cells might also be a problem in humans needs to be addressed in future studies.

In summary, we established an efficient protocol for expansion and transduction of primary human NK cells with BaEV-pseudotyped CAR lentiviral vectors, which allow enrichment of the CAR NK cells to high purities. We also developed a simple method for blocking activation receptor-dependent killing of target cells by NK cells, thereby paving the way for successful evaluation of CAR NK cells targeting a variety of antigens on leukemic blasts and also on other malignant cells in future studies. Since the MACS enrichment protocol can easily be established under GMP conditions, our study seems to be highly informative for the *off-the-shelf* manufacturing of CAR NK cells for clinical use in cellular immunotherapy.

## Data Availability Statement

The original contributions presented in the study are included in the article/supplementary material. Further inquiries can be directed to the corresponding author.

## Ethics Statement

The use of peripheral blood from healthy donors was approved by the local ethics committee (Heinrich Heine University, Düsseldorf, protocol #2019-623).

## Author Contributions

MS, AB, CH, NB, MU, CW, DR, NN, and HH planned the experiments. MS, AB, CH, AThi, SCC, NB, SS, ATho, DS, MH, and NN conducted the experiments. MS, AB, CH, AThi, SCC, SS, NB, ATho, DS, MH, MU, KS, CM, CW, DR, NN, and HH critically analyzed data. MS, AB, CH, NN, and HH wrote the manuscript with the help of the other authors. All authors approved the final manuscript.

## Funding

This work was supported, in part, by funding from the Medical Research School Düsseldorf, DSO, Heinrich Heine University, Düsseldorf; the Essener Elterninitiative zur Unterstützung krebskranker Kinder e.V.; and the UMEA Clinician Scientist Program of the Medical Faculty of the University of Duisburg-Essen funded by the Deutsche Forschungsgemeinschaft (DFG) and within the framework of the iCAN33 project, funded by the European Regional 470 Development Fund NRW (ERDF, German EFRE) 2014-2020. NB and CM acknowledge financial support from the DFG through the SFB1208 program “Identity and Dynamics of Membrane Systems” (A12). CM acknowledges financial support *via* the “Freigeist fellowship” of the Volkswagen Foundation.

## Conflict of Interest

HH and CW are inventors on a patent describing the CD34 hinge for CAR immune effector cells.

The remaining authors declare that the research was conducted in the absence of any commercial or financial relationships that could be construed as a potential conflict of interest.

## Publisher’s Note

All claims expressed in this article are solely those of the authors and do not necessarily represent those of their affiliated organizations, or those of the publisher, the editors and the reviewers. Any product that may be evaluated in this article, or claim that may be made by its manufacturer, is not guaranteed or endorsed by the publisher.
